# Prescription of potentially inappropriate medications in elderly outpatients: a survey using 2015 Japanese Guidelines

**DOI:** 10.1007/s11096-020-00967-9

**Published:** 2020-01-21

**Authors:** Keiko Fujie, Risa Kamei, Risa Araki, Koichi Hashimoto

**Affiliations:** 1 Department of Clinical and Translational Research Methodology, Faculty of Medicine, University of Tsukuba, 1-1-1 Tennodai, Tsukuba, Ibaraki 305-8575 Japan; 2 Graduate School of Comprehensive Human Sciences, University of Tsukuba, 1-1-1 Tennodai, Tsukuba, Ibaraki 305-8575 Japan

**Keywords:** Dispensing pharmacy, Guideline, Japan, Outpatient, Polypharmacy, Potentially inappropriate medication

## Abstract

*Background* In recent years, rapid increase of elderly population has become a major social problem in developed countries. They tend to receive an increasing number of prescibed drugs due to multiple illnesses, which might include inappropriate medications, in turn leading to health hazards and rising healthcare cost. *Objective* To evaluate the current status of potentially inappropriate medications prescribed for elderly outpatients and filled by dispensing pharmacies using the recent Japanese Guidelines, and to determine factors that are related to prescribing potentially inappropriate medications. *Setting* A cross-sectional study of older patients (≥ 75 years) who visited dispensing pharmacies in the Ibaraki Prefecture, Japan. *Method* We identified patients prescribed potentially inappropriate medications using the “List of Medications that Require Particularly Careful Administration” in the Guidelines (Guideline List). We explored patient’s factors related to polypharmacy (≥ 5 medications) and prescription of inappropriate medications through multivariate analysis, and a cutoff value for predicting potentially inappropriate medications through receiver operating characteristic curve analysis. *Main outcome measure* Prevalence of polypharmacy and potentially inappropriate medications, and patient’s factors associated with them. *Results* Of 8080 patients (39,252 medications) who visited pharmacies during the study period, 43.1% (3481) were prescribed ≥ 5 medications. In total, 2157 patients (26.7%) were prescribed at least one potentially inappropriate medication. The most prescribed inappropriate medication class was (benzodiazepine) sedatives and hypnotics. Potentially inappropriate medications were 7.11 times (95% CI 6.29–8.03) and 1.51 times (1.34–1.71) more likely to be prescribed for patients with ≥ 5 medications and those prescribed by multiple physicians, respectively. A cutoff value for potentially inappropriate medications was found to be five for the total number of medications and four for the number of chronic medications with a systemic effect. *Conclusion* Prescription of potentially inappropriate medications was increased among patients with ≥ 5 medications and those chronically prescribed ≥ 4 medications with a systemic effect. The Guideline List should be actively used to screen such patients, and to carefully examine prescriptions. Particular care should be exercised when patients are visiting multiple physicians.

## Impacts on practice

- To prevent polypharmacy and PIMs, it is necessary for elderly outpatients to avoid being prescribed medication by multiple physicians as much as possible.
- Pharmacists at dispensing pharmacies and physicians must be encouraged to proactively check if there are PIMs in the prescriptions when elderly outpatients are prescribed ≥ 5 medications or ≥ 4 chronic-phase systemic drugs.
- Physicians are expected to regularly review the prescriptions for the elderly, and to take particular care to prescribe bennzodiazepine sedatives and hypnotics.

## Introduction

In recent years, the rapidly increasing number of elderly people in the Japanese population has become a major social problem. The World Health Organization (WHO) defines societies with an aging rate (the percentage of the total population that is aged ≥ 65 years) > 21% as “super-aged societies” [1]. With an aging rate of 28.1% in 2018 [2], Japan is categorized as a super-aged society. The aging of society is not limited to Japan and other developed countries, which is also the case even in developing countries in Asia [1]. As people age, they tend to take an increasing number of medical treatments including drug prescription, which may lead to health hazards. The elderly people are susceptible to adverse drug events (AEs) [3] for two main reasons; the first being “polypharmacy”, which is generally considered to be a situation in which a patient is taking five or more medications, is more common due to the increasing number of co-morbidities [4]. As reported [5–7], the cutoff of five medications is based on the increased risk of falling, frailty, and death among the elderly. Other studies have reported that increase in the number of prescribed drugs elevates the risk of being prescribed potentially inappropriate medications (PIMs) and of developing AEs [8–11]. The second reason is because people’s ability to metabolize drugs is decreased as they age due to physiological changes including reduced liver and renal functions [12]. Drug sensitivity is also increased in elderly people; even small doses of a drug can lead to AEs. For the abovementioned reasons, reducing the number of unnecessary and inappropriate prescriptions for the elderly is a major issue in clinical settings. Studies have suggested that polypharmacy leads to AEs due to drug interactions, dispensing errors, and errors associated with medicine use, but is also associated with underuse [13, 14]. Thus, this is an issue that is related to all aspects of the appropriateness of prescriptions.

Given that these background circumstances are common in advanced nations of the world, the safety of the pharmacotherapy provided to the elderly has come to be recognized as an important global problem. This has led to submission of the Beers Criteria [15, 16] and the screening tool of older people’s prescriptions (STOPP)/screening tool to alert to right treatment (START) Criteria [17, 18]. In Japan, the *Guidelines for the Safety of Pharmacotherapy in the Elderly* was released in 2015 (GL2015) [12]. These guidelines were directed to the elderly aged ≥ 75 years, who are “old–old” in the Japanese medical insurance system. The GL2015 comprises a “List of Medications that Require Particularly Careful Administration” which indicates PIMs, and a “List of Medications that should be Considered for Administration” which is designed to eliminate underuse. Despite the necessity of reducing PIMs, there is still little data on polypharmacy and PIMs among elderly patients in Japan, resulting in insufficient appreciation of this problem among healthcare professionals that has direct contact with patients’ prescriptions.

## Aim of the study

The aim of this study was to evaluate the current status of polypharmacy and PIMs among elderly outpatients whose prescriptions were filled by dispensing pharmacies in Japan, and to find patient’s factors associated with prescription of PIMs.

## Ethics approval

The study protocol was approved by the Medical Institutional Review Boards of the University of Tsukuba. This study utilized anonymized data from dispensing pharmacies. Information regarding this study was disclosed on the homepage of our research laboratory.

## Method

### Subjects and setting

We conducted a retrospective cross-sectional study. The study subjects were elderly outpatients aged ≥ 75 years whose prescriptions were filled at dispensing pharmacies in urban and suburban areas of Ibaraki Prefecture, Japan. The number of targeted pharmacies throughout the prefecture was 12, nine of which were located in the vicinity of regional core hospitals. Anonymized data from the pharmacies included the following: pharmacy, prescription number, patient identification number, age, sex, name and number of beds of prescribing medical facility, prescribing medical department, brand name, content and price of each medication, and number of days the medication was prescribed for. The following patient background data were unavailable: history of the current illness, complications, and laboratory test data.

In Japan, mostly, patients with chronic diseases are seen at the outpatient department once a month. To prevent repeated use of the same patient’s data and to account for the possibility that some patients may visit the outpatient department at intervals longer than 30 days, we extended the study period by 10 days (total of 40 days) from February 1 to March 12, 2015 (the period with the fewest number of single and consecutive holidays in the year). Age of the subject was defined as the age on the first prescription during the study period, except that patients who turned 75 years during the period were regarded as 75 years. All subjects aged ≥ 85 years were listed as “85” in the data.

### Definition of prescription and polypharmacy

We considered that prescriptions from the same medical department at the same medical facility were given by the same physician. In patients with ≥ 2 prescriptions during the study period, only the data of the first prescription by the same physician were utilized. Thus, the number of prescriptions for a single patient matched the number of physicians who examined the patient. Prescriptions written by different physicians were regarded as those for different medications, and therefore each medication was included in the total number of medications for the patient. “Polypharmacy” was defined as cases in which the total number of medications was ≥ 5 [3–5]. We also counted “chronic-phase systemic drugs”, which included oral, self-injectable, and systemic external drugs (designed to be absorbed into the bloodstream), and excluded “acute-phase prescriptions (≤ 7 days)”.

### Screening for PIMs

In this study, “the List of Medications that Require Particularly Careful Administration” in GL2015 (STOPP-J) [12], which includes medications intended for long-term administration, was used for PIMs screening. The drugs in the list are organized into 20 categories and 29 classes. Specifically, only the drug classes, which could be clearly determined from the data to correspond to medications that they “should not be used” or that their “use should be kept to the minimum possible”, were targeted for screening. Drug classes with recommended administration period or doses were excluded from screening because such data were not available. Thus, 11 categories and 13 classes of drugs were adopted in this study (Table 1). We investigated the generic names of the compounds in each drug class and subsequently searched for their brand names using the Kyoto Encyclopedia of Genes and Genomes (KEGG) DRUG Database (www.genome.jp/kegg/).

**Table 1:** Drug classes from the “List of Medications that Require Particularly Careful Administration (STOPP-J)” that were screened in this study

Category	Drug class	Recommended use
Sleeping drugs	Benzodiazepine-based sleeping drugs and anti-anxiety drugs	Do not use long-lasting effect type and triazolam Use the other benzodiazepines as little as possible
Anti-depressants	Tricyclic anti-depressants	Use as little as possible
Sulpiride	Sulpiride	Use as little as possible
Anti-Parkinson drugs	anti-Parkinson drugs	Use as little as possible
Alpha blockers	Receptor subtype non-selective α_1_ receptor blockers	Use as little as possible
First generation H_1_ blockers	First generation H_1_ blockers	Use as little as possible
H_2_ blockers	H_2_ blockers	Use as little as possible
Antiemetics	antiemetics	Use as little as possible
Anti-diabetes drugs	Sulfonylurea	Do not use when possible
	Biguanide	Do not use when possible
	SGLT2 inhibitors	Use as little as possible
Insulin	Sliding scale insulin	Use as little as possible
Overactive bladder drugs	Oxybutynin	Use as little as possible

*SGLT2* sodium-glucose co-transporter 2

Using the brand names identified above, we screened for PIMs among the drugs included in the prescriptions. For each drug category, we indicated the total numbers of relevant drugs, prescriptions including PIMs, and patients prescribed PIMs. In situations with multiple categories of PIMs in the same prescription for a patient, we considered that as an overlap, and also counted the actual number of prescriptions and patients after removing the overlapping numbers.

### Statistical analysis

We calculated the median, interquartile range (IQR), and maximum values of the number of medications for each patient. In order to identify patient’s factors that are related to polypharmacy, the Chi square test for categorical variables and the Mann–Whitney U test for continuous variables were performed, with statistical significance set at *P *< 0.05 on both sides. We also performed multivariate logistic regression analysis, calculated the adjusted odds ratio (aOR) and the 95% confidence interval (CI) for each factor, and investigated their relationship with polypharmacy. The results of PIM screening were included in the descriptive analysis and used to determine the factors related to PIMs as described for polypharmacy.

We performed receiver operating characteristic curve (ROC) analysis in order to determine whether the number of prescribed drugs is a useful indicator in predicting the presence of PIMs, and calculated the Youden Index [19] to search for the cutoff values for PIMs.

Data processing and analysis was performed using the Microsoft Excel 2016 and SPSS Statistics Version 24, respectively.

## Results

There were 8080 patients aged ≥ 75 (45.4% male) years who had their prescriptions filled at the target pharmacies over the 40-day study period. The patient background characteristics are shown in Table 2. The percentage of patients aged ≥ 85 years was 25.6%. The number of patients who were examined by ≥ 2 physicians was 1829 (22.6%). The maximum number of physicians who prescribed for a patient was six. The most common departments where patients received prescriptions were neurosurgery (17.1%) and general internal medicine (16.3%). Nearly half of the prescriptions were issued from relatively small medical facilities with ≤ 99 beds.

**Table 2:** Patient background characteristics and total number of medications

	Total no. of patients n = 8080
Sex, n (%)	
Male	3668 (45.4)
Female	4412 (54.6)
Age, n (%), years	
75–84	6009 (74.4)
85 and above	2071 (25.6)
No. of prescribing physicians per patient, n (%)	
1	6251 (77.4)
2	1365 (16.9)
3	361 (4.5)
4	77 (1.0)
5	20 (0.2)
6	6 (0.1)
Departments, n (%)^a^	
Neurosurgery	1795 (17.1)
General internal medicine	1715 (16.3)
Orthopedic surgery	1358 (12.9)
Ophthalmology	1322 (12.6)
Cardiovascular medicine	883 (8.4)
Others	3435 (32.7)
Bed no. of medical facilities, n (%)^a^	
300 and more	3518 (33.5)
100–299	2075 (19.7)
99 and less	4915 (46.8)
No. of prescribed drugs per patient, median (IQR), [max]	
Overall	4 (2–7) [28]
Chronic-phase systemic drugs	3 (1–6) [25]

*IQR* interquartile range

^a^n = 10,508 was the total number of prescriptions, including multiple prescriptions per patient

The total 39,252 medications after exclusion of at least the second prescription by the same physician were included in our analyses. Among the overall total of medications, there were 32,313 oral drugs, 192 self-injectable drugs, 239 systemic external drugs, and 1949 acute-phase internal drugs, resulting in 30,795 (78.5%) chronic-phase systemic drugs. The median (IQR) and maximum values for the numbers of medications for each patient are shown in Table 2. The maximum numbers of overall medications and chronic-phase systemic medications were 29 and 25, respectively.

Overall, 43.1% (3481/8080) patients had polypharmacy. At univariate analysis, those aged ≥ 85 years, and those who were prescribed by ≥ 2 physicians had significantly higher percentages of polypharmacy (*P *< 0.001). No statistically significant difference was found between the sexes (Table 3). Our multivariate logistic regression analysis on polypharmacy showed that being aged ≥ 85 years had the aOR of 1.37 (95% CI 1.23–1.53) against aged < 85 years, while being prescribed by one additional physician had the aOR of 5.73 (95% CI 5.11–6.42) (Table 3).

**Table 3:** Analysis of factors related to polypharmacy

	Univariate analysis			Multivariate analysis	
	Non-polypharmacy (< 5 medications) n = 4599	Polypharmacy (≥ 5 medications) n = 3481	*P*^a^	Adjusted OR^b^ (95% CI)	*P*^c^
Sex, female, n (%)	2484 (54.0)	1928 (55.4)	0.219	1.02 (0.92–1.12)	0.739
Age, ≥ 85, n (%)	1065 (23.2)	1006 (28.9)	<0.001	1.37 (1.23–1.53)	< 0.001
No. of prescribing physicians per patient, ≥ 2, n (%)	385 (8.4)	1444 (41.5)	<0.001	5.73 (5.11–6.42)^d^	< 0.001

Adjusted confounding variables in the multivariate logistic regression analysis; sex, age and the number of physicians per patient

*OR* odds ratio, *CI* confidence interval

^a^*P* value according to Chi square test

^b^Odds ratio adjusted for sex, age, number of prescribing physicians per patient

^c^*P* value according to the logistic regression analysis

^d^Number of prescribing physicians per patient was treated as a continuous variable at multivariate analysis

The results of PIMs screening using the STOPP-J are shown in Table 4. Among 39,252 medications included in the analyses, 2905 (7.4%) were inappropriately prescribed. Our analysis also indicated that 21.6% (2273/10,508) prescriptions included ≥ 1 PIMs, and that 26.7% (2157/8080) patients were given prescriptions with ≥ 1 PIMs. As shown in Table 4, 1460 (50.3%), 632 (21.8%), and 316 (10.9%) of the PIMs were sleeping drugs, H2-receptor antagonists, and diabetes drugs, respectively. When divided into two groups as non-polypharmacy (< 5 medications) and polypharmacy (≥ 5 medications), PIMs were mostly sleeping drugs (non-polypharmacy: 51.9%, polypharmacy: 49.9%), H2-receptor antagonists (23.0%; 21.5%), and diabetes drugs (6.9%, 11.8%), in that order. Of all the patients with PIM, 27.0% (583/2,157) were prescribed with multiple PIMs, and the maximum number of PIMs per patient was six.

**Table 4:** Results of PIMs screening based on the STOPP-J

Category of PIMs	Medications qualified as PIMs, n (%)	Prescriptions including PIMs, n (%)	Patients prescribed PIMs, n (%)
Sleeping drugs	1460 (50.3)	1276 (48.5)	1251 (48.2)
Antidepressants	27 (0.9)	27 (1.0)	27 (0.3)
Sulpiride	21 (0.7)	21 (0.8)	20 (1.0)
Anti-Parkinson drugs	6 (0.2)	6 (0.2)	6 (0.2)
Alpha blockers	221 (7.6)	217 (8.3)	216 (8.3)
First-generation H1 blockers	17 (0.6)	17 (0.6)	16 (0.6)
H2 blockers	632 (21.8)	632 (24.0)	628 (24.2)
Antiemetics	35 (1.2)	35 (1.3)	35 (1.3)
Diabetes drugs	316 (10.9)	261 (9.9)	260 (10.0)
Insulin	160 (5.5)	128 (4.9)	127 (4.9)
Overactive bladder drugs	10 (0.3)	10 (0.4)	10 (0.4)
Total	2905	2630^a^ (2273)^b^	2596^a^ (2157)^b^

*PIM* potentially inappropriate medication, *STOPP-J* list of medications that require particularly careful administration

^a^Sum of each category

^b^Actual number after eliminating overlaps

In the univariate analysis comparing patients with or without PIMs, a significant difference was found in sex and the number of physicians who prescribed for a patient. The numbers of overall and chronic-phase systemic medications were also significantly associated with PIMs (Table 5). In the multivariate analysis, polypharmacy (aOR: 7.11, 95% CI 6.29–8.03) and multiple physicians prescribing for a patient (1.51, 1.34–1.71) showed significant relationship with prescription of PIMs. Age was not found to be significantly related to PIMs before and after adjustment (Table 5).

**Table 5:** Analysis of factors related to PIMs

	Univariate analysis			Multivariate analysis	
	PIMs = No n = 5923	PIMs = Yes n = 2157	*P*^a^	Adjusted OR^b^ (95% CI)	*P*^c^
Sex, female, n (%)	3192 (53.9)	1220 (56.6)	0.033	1.10 (0.99–1.23)	0.084
Age, ≥ 85, n (%)	1485 (25.1)	586 (27.2)	0.056	0.96 (0.85–1.08)	0.486
No. of prescribing physicians per patient, ≥ 2, n (%)	989 (16.7)	840 (38.9)	< 0.001	1.51 (1.34–1.71)	< 0.001
Polypharmacy	1800 (30.4)	1681 (77.9)	< 0.001	7.11 (6.29–8.03)	< 0.001
No. of drugs, median (IQR)					
Overall	3 (2–5)	7 (5–10)	< 0.001^d^		
Chronic-phase systemic drugs	2 (1–4)	6 (4–9)	< 0.001^d^		

Adjusted confounding variables in the multivariate logistic regression analysis; sex, age, the number of physicians per patient and polypharmacy

*PIM* potentially inappropriate medication, *OR* odds ratio, *CI* confidence interval, *IQR* interquartile range

^a^*P* value according to Chi square test

^b^Odds ratio adjusted for sex, age, number of prescribing physicians per patient, and polypharmacy

^c^*P* value according to logistic regression analysis

^d^*P* value according to Mann–Whitney U test

**Fig. 1:**
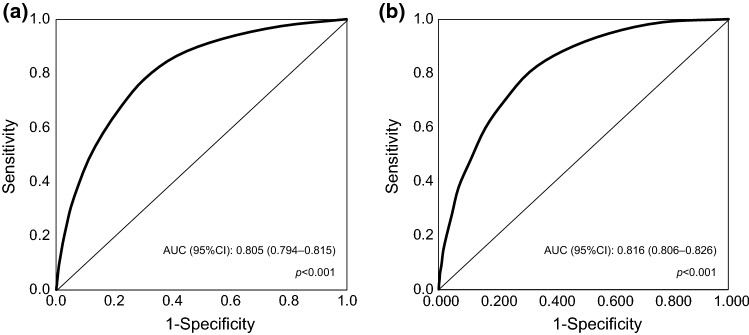
ROC curves for no. of drugs and PIMs. **a** Overall no. of drugs. **b** No. of chronic-phase systemic drugs. AUC, area under the curve; CI, confidence interval; ROC, receiver operating characteristics curve; PIM, potentially inappropriate medication

The ROC, area under the curve (AUC), AUC *P* value, and 95% CI for PIMs are shown in Fig. 1. The AUCs for both the overall number of medications and the number of chronic-phase systemic drugs exceeded 0.8 (*P* < 0.001). Thus, we estimated that these numbers have predictive ability for PIMs. The results indicated that the Youden Index reached a maximum value at 4.5 and 3.5, respectively. The ideal cutoff value for PIMs was found to be five for the overall number of medications and four for the number of chronic-phase systemic drugs.

## Discussion

In this study, we elucidated the current status of polypharmacy and PIMs based on the STOPP-J among the elderly outpatients in Japan. The results indicated that 43.1% of outpatients aged ≥ 75 years experienced polypharmacy (≥ 5 medications), and that 26.7% of them had at least one PIM.

Several previous studies [3, 5–8] reported that, with increasing number of drugs prescribed, increased risk of drug interactions and drug duplications occurred, which subsequently increased the likelihood of AEs. Since many elderly people have multiple illnesses [5], prescriptions of long-term multiple drugs are inevitable. Furthermore, elderly people have reduced hepatic and renal functions which in turn make the experience of AEs more likely [20]. It is therefore advisable to reduce the number of medications prescribed for the elderly to the minimum required limit.

We found that 22.6% of the patients had been prescribed by ≥ 2 physicians. The results of our multivariate analysis indicated that the risk of experiencing polypharmacy increased by 5.73 times (95% CI 5.12–6.42) each time the patient was prescribed by one additional physician. In general, the number of illnesses a person suffers from increases with aging, and there is a tendency for people to visit more physicians and to be prescribed more medications. Prescriptions by multiple physicians would make it difficult for them to keep track of the medications being utilized by the patient. In addition, since it is difficult for the elderly to self-manage their drugs [21], they are more likely to experience AEs as the number of prescribing physicians increases [22]. To prevent AEs, physicians are required to prescribe new medications only after a thorough review of the medications being used by the elderly.

The largest percentage of drugs that qualified as PIMs were sleeping drugs (50.3%). Since sleep disorders are more common as people age, there is a tendency for more sleeping drugs corresponding to PIMs to be prescribed to the elderly [23]. Benzodiazepines, the most commonly used sleeping medication, are known to increase the risk of falling among the elderly, therefore, it is recommended that a different sleeping drug be prescribed whenever possible [24, 25]. However, since benzodiazepines are habit-forming, it is often difficult for patients to stop their use immediately. It is effective to gradually reduce the dose while monitoring the patient’s condition to ensure that withdrawal symptoms do not arise, until switching to another drug [24, 26].

Our multivariate analysis indicated that polypharmacy and being prescribed by multiple physicians were significantly related to PIMs. In addition, PIMs were significantly related to the number of prescribed drugs. As reported in previous studies [5–8], larger numbers of prescribed drugs increase the risk of PIMs, which is consistent with the present results. It was shown that 78.5% of medications prescribed to the elderly were long-term systemic medications; thus, it is important to pay more attention to such drugs. The results of our ROC analysis indicated that the cutoff values for PIMs for the overall number of drugs and for chronic-phase systemic drugs were five and four, respectively. This suggests that once these numbers are exceeded, there is a higher risk of PIMs. Therefore, in clinical settings, a careful examination and re-evaluation of the prescriptions issued to elderly patients aged ≥ 75 years exceeding these cutoffs should be proactively conducted.

As the GL2015 is mainly concerned with safety, unlike treatment guidelines, prescribing drugs that qualify as PIMs may not necessarily be inappropriate. However, the drugs included in the STOPP-J have a high risk of causing AEs in the elderly; therefore, it is recommended that they should not be prescribed as far as possible, or that alternative medications be utilized. Recently another Japanese group also reported the current status of polypharmacy and PIMs in elderly people who visited dispensing pharmacies [27], however, they used the former edition of STOPP-J. In addition, they did not take multiple prescriptions for an elderly patient into consideration and not show a cutoff value of the number of drugs for predicting PIMs. We believe that the results of this study will contribute to increased propriety in prescriptions issued to elderly patients by indicating criteria to detect PIMs, and we expect that pharmacists at dispensing pharmacies play a role in the improvement of the prescriptions. Several studies have shown the efficacy of pharmacists’ assessment and intervention on appropriate medications in hospitalized patients [11, 28]. The similar trials are required to be conducted in elderly outpatients for the next step.

The present study had several limitations. First, since the study period was during winter, it is possible that the prescribed drugs may have some season-related differences. In order to investigate the possibility, we compared our data with data from the period between June 1 and July 10, 2015, resulting in no notable differences in the trends for polypharmacy and PIMs. Second, our prescription data were obtained from dispensing pharmacies in one prefecture, which might limit the generalization of our results to entire population of elderly people in Japan. However, we incorporated data from the whole geographical region and medical facilities into our analyses; therefore, we believe the results reflect real-world status of prescriptions in general elderly population. Finally, a patient who receives a prescription at an outpatient department might not always visit the same dispensing pharmacy, which suggests the possibility that we could not collect all prescriptions for a patient. In addition, we screened for PIMs for only drug classes which do not require other information than the drug name this time. Thus, when taking into consideration the administration period and dose of the drugs, it is predictable that a larger number of medications would have qualified as PIMs. Therefore, our results are likely an underestimation of the actual number of medications and PIMs.

## Conclusion

The results of this study confirmed that polypharmacy is prevalent among elderly people, and that polypharmacy increased the risk of PIMs. It is recommended that proactive prescription screening using the STOPP-J be conducted in cases in which the overall number of drugs exceeds five, or the number of chronic-phase systemic drugs exceeds four. The results also indicated that being prescribed by multiple physicians was related to PIMs. Thus, it is necessary to proactively reduce the number of physicians who examine an elderly patient and to ascertain each patient’s drug administration status at examinations.
